# From the archives: Plasmodesmata—regulation of function, targeting by pathogenic bacteria, and plasmodesmal-associated proteins

**DOI:** 10.1093/plcell/koaf040

**Published:** 2025-03-03

**Authors:** Mariana Schuster

**Affiliations:** Assistant Features Editor, The Plant Cell, American Society of Plant Biologists; Leibnitz Institute of Plant Biochemistry, Halle (Saale) 06120, Germany

## 2024: Regulation of plasmodesmal function

Plasmodesmata (PD) are membrane-lined channels that connect the cytoplasm, endoplasmic reticulum, and plasma membrane of neighboring plant cells. These cell-to-cell connections facilitate the exchange of molecules, enabling the transfer of information and resources. The PD aperture, which determines the size of the molecules that can pass from one cell to another, is regulated by the deposition and degradation of the polysaccharide callose (β-1,3-glucan) within the cell walls adjacent to these channels. PLASMODESMATA-LOCATED PROTEINS (PDLPs) are integral to callose homeostasis and, consequently, to the regulation of PDs. Li et al. (2024) investigated the function of PDLPs in Arabidopsis (*A. thaliana*) through overexpression followed by phenotypic characterization. Overexpression of PDLP5 and PDLP6 resulted in delayed growth and excessive starch accumulation in mature leaves. Remarkably, the starch overaccumulation observed in the PDLP5 and PDLP6 overexpression lines exhibited distinct, cell type–specific patterns. Single-cell RNA sequencing and reporter lines revealed that PDLP5 is expressed in epidermal and cortical cells, whereas PDLP6 expression was predominantly observed in the vasculature. Given the distinct patterns of starch accumulation and the differential expression of PDLP5 and PDLP6, the authors investigated plasmodesmal function in the overexpression lines at specific cell interfaces. Increased callose deposition and restricted molecular movement were observed in the stomata of cells expressing each PDLP, which explained the corresponding starch accumulation patterns. Furthermore, SUCROSE SYNTHASE 6 (SUS6) and CALLOSE SYNTHASE 7 (CALS7) were identified as interacting partners of both PDLPs using in vitro pull-down assays. Introduction of either sus*6* or *cals7* mutation in the PDLP6 overexpression line resulted in suppressed starch hyperaccumulation. The authors concluded that SUS6 and CALS7 are required for the function of PDLP6 in PDs.

## 2020: Targeting of plasmodesmata by pathogenic bacteria

Plants close PDs as part of their immune response. The expression of PLASMODESMATA-LOCATED PROTEIN 5 (PDLP5) is upregulated during pathogen perception, suggesting a key role for this protein in regulating PD function in the context of disease. To overcome plant defense responses, pathogens secrete specialized proteins known as effectors. Aung et al. (2020) identified and characterized a PD-associated effector from *Pseudomonas syringae* pv. *tomato* (*Pst*) DC3000, termed HopO1-1. Using Arabidopsis transgenic lines expressing a fluorescently tagged version of HopO1-1, the authors demonstrated that HopO1-1 specifically localizes to PDs. This effector contains an ADP-ribosyltransferase (ADP-RT) domain, and Aung et al. (2020) confirmed its mono-ADP-ribosyltransferase (mADP-RT) activity. Plants expressing HopO1-1 exhibited increased intercellular movement of YFP molecules, indicating enhanced PD permeability. Co-immunoprecipitation and bimolecular fluorescence complementation assays further revealed that HopO1-1 interacts with both PDLP5 and PDLP7. Moreover, the authors showed that HopO1-1 affects the stability of PDLP5 and PDLP7 via a proteasome-dependent mechanism. Finally, ΔhopO1-1 mutant strains of Pst DC3000 displayed reduced virulence, characterized by impaired colonization of cells surrounding the infection site compared with the wild-type strain (Figure). Notably, the authors showed that the contribution of HopO1-1 to virulence depends on its PD-specific localization and mADP-RT activity.

**Figure.**
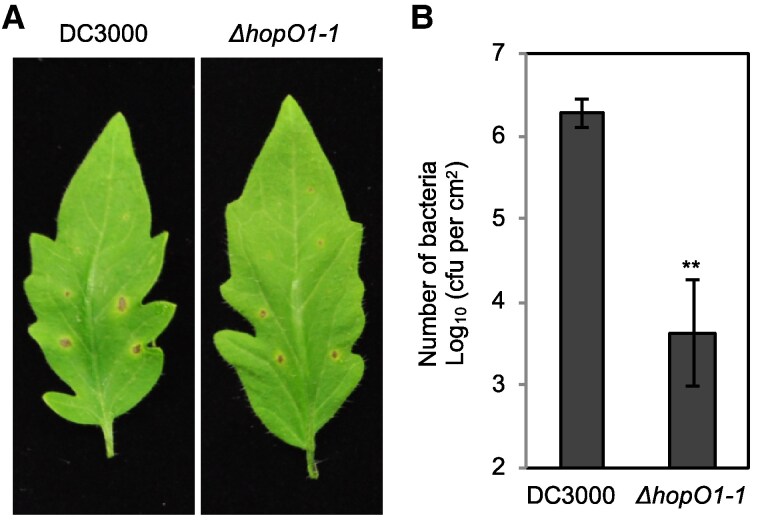
HopO1-1 is crucial for colonization of *P. syringae* Surrounding Infection Sites. **A)** Disease phenotypes of tomato leaves after local infection with *Pst* DC3000 and the *ΔhopO1-1* mutant using a needle. The images were taken 7 days after infection. **B)** Bacterial multiplication was determined 7 d after infection by counting the bacterial number (cfu/cm^2^ leaf area). The needle infection sites were removed using a biopsy punch (2 mm radius), and the distal tissues were collected to determine bacterial growth. Error bars represent SE from 6 samples. Statistical differences between DC3000 and *ΔhopO1-1* are analyzed with a 2-tailed *t* test (**P < 0.005). Reprinted from Aung et al. (2020), Figure 8.

## 2005: Plasmodesmal-associated proteins

The identification of PD-associated proteins is a critical step toward elucidating the function of these essential cellular structures. Sagi et al. (2005) reported SE-WAP41 as the first PD-associated protein. SE-WAP41 was isolated from maize (*Zea mays*) and identified using an antiserum known to label PDs. The protein was characterized through microsequencing and subsequent transcript amplification with degenerate primers. The cloned SE-WAP41 sequence corresponded to a gene encoding a class 1 reversibly glycosylated polypeptide (^C1^RGP). Heterologous production of SE-WAP41 facilitated the generation of specific antibodies, demonstrating that SE-WAP41 is enriched in cell wall fractions containing PDs. Localization to PDs was further validated by transient expression of a GFP-tagged version of the Arabidopsis homolog (AtRGP2) in *Nicotiana tabacum* and the development of fluorescent AtRGP lines. This study provided the foundation for further investigation into PD-associated proteins and their roles in intercellular communication.

## Data Availability

There are no new data associated with this article.

## Dive Curated Terms

The following phenotypic, genotypic, and functional terms are of significance to the work described in this paper:

PDLP7 Gramene: AT5G37660
PDLP7 Araport: AT5G37660

SUS6 Gramene: AT1G73370
SUS6 Araport: AT1G73370

PDLP6 Gramene: AT2G01660
PDLP6 Araport: AT2G01660

PDLP5 Gramene: AT1G70690
PDLP5 Araport: AT1G70690

CALS7 Gramene: AT1G06490
CALS7 Araport: AT1G06490
